# Integrative landscape analysis of prognostic model biomarkers and immunogenomics of disulfidptosis-related genes in breast cancer based on LASSO and WGCNA analyses

**DOI:** 10.1007/s00432-023-05372-z

**Published:** 2023-09-22

**Authors:** Shuyan Liu, Yiwen Zheng, Shujin Li, Yaoqiang Du, Xiaozhen Liu, Hongchao Tang, Xuli Meng, Qinghui Zheng

**Affiliations:** 1 The Second Clinical Medical College, Zhejiang Chinese Medical University, Hangzhou, 310053 Zhejiang China; 2 Department of Breast Surgery, General Surgery, Cancer Center, Zhejiang Provincial People’s Hospital (Affiliated People’s Hospital), Hangzhou Medical College, Hangzhou, 310053 Zhejiang China; 3 Key Laboratory for Diagnosis and Treatment of Upper Limb Edema and Stasis of Breast Cancer, Hangzhou, 310053 Zhejiang China; 4 Laboratory Medicine Center, Zhejiang Provincial People’s Hospital (Affiliated People’s Hospital, Hangzhou Medical College), Hangzhou, 310053 Zhejiang China

**Keywords:** Breast cancer, Disulfidptosis, Tumor-infiltrating immune, Tumor mutation burden, Immunotherapy

## Abstract

**Background:**

Disulfidptosis is a novel type of programmed cell death. However, the value of disulfidptosis-related genes (DRGs) in the prediction of breast cancer prognosis is unclear.

**Methods:**

RNA-seq data of 1231 patients, together with information on patient clinical characteristics and prognosis, were downloaded from TCGA. DRGs were identified between cancerous and non-cancerous tissues. The LASSO algorithm was used to assign half of the samples to the training set. Risk scores were used for construction of a prognostic model for risk stratification and prognosis prediction, and the clinical applicability was examined using a line diagram. The relationships between risk scores, immune cell infiltration, molecular subtypes, and responses to immunotherapy and chemotherapy were examined.

**Results:**

We identified and obtained four DRG-related prognostic lncRNAs (AC009097.2, AC133552.5, YTHDF3-AS1, and AC084824.5), which were used for establishing the risk model. Longer survival was associated with low risk. The DRG-associated lncRNAs were found to independently predict patient prognosis. The AUCs under the ROCs for one-, three-, and 5-year survival in the training cohort were 0.720, 0.687, and 0.692, respectively. The model showed that the high-risk patients had reduced overall survival as well as high tumor mutation burdens. Furthermore, high-risk patients showed increased sensitivity to therapeutic drugs, including docetaxel, paclitaxel, and oxaliplatin.

**Conclusion:**

The risk score model was effective for predicting both prognosis and sensitivity to therapeutic drugs, suggesting its possible usefulness for the management of patients with breast cancer.

**Supplementary Information:**

The online version contains supplementary material available at 10.1007/s00432-023-05372-z.

## Introduction

The global levels of breast cancer are rising steadily. The disease is associated with high rates of mortality (Siegel et al. 2018, 2020; DeSantis et al. 2019) together with accounting for approximately one-third of new tumors in women (DeSantis et al. 2019; Sung et al. 2021). Recent advances in new drugs and therapeutic targets have led to improvements in both the treatment and prognosis of breast cancer (Crozier et al. 2020; Voorwerk et al. 2019; Dirix et al. 2018). Current treatment modalities include surgery, radio-, chemo-, and hormone therapy, as well as targeted therapy (Esteva et al. 2019). The great burden of breast cancer on the global economy and healthcare system indicates the urgency of understanding its pathogenesis. The ability to rapidly diagnose and treat breast cancer would be highly beneficial in improving patient outcomes (Rakha and Pareja 2021). Thus, the identification of novel markers and the construction of models for prognosis prediction would be of great value.

Disulfidptosis is a novel form of programmed cell death. Disulfidptosis occurs in cells with low glucose and high SLC7A11 levels and is characterized by abnormal disulfide bonding between cytoskeletal proteins, specifically actin. This results in structural damage within the cell, ultimately causing the destruction of the actin cytoskeleton and cellular death (Zheng et al. 2023; Machesky 2023). This important finding is expected to aid the discovery of new prognostic markers to stimulate disulfide induction therapy for cancers that are insensitive to other treatments and apoptosis-resistant. The elevated expression of SLC7A11 suggests the possibility of a therapeutic window characterized by the inhibition of glucose transporters and disulfide bond formation, which might allow the specific treatment of cancer cells without affecting normal cells (Liu et al. 2023).

It appears likely that disulfidptosis is involved in the tumorigenesis of various cancers, suggesting its potential as a marker for both diagnosis and treatment. However, to date, there is no information on disulfidptosis in breast cancer. The present study, thus disulfidptosis in breast cancer, is focusing especially on the tumor microenvironment. LASSO was used for identifying disulfidptosis-related genes (DRGs) linked with tumor prognosis, using 1231 patient samples. A DRG-scoring method was developed for the assessment of overall survival (OS), and the effectiveness of the model for assessing immune cell infiltration, the tumor mutation burden (TMB), mutational status, and drug resistance was evaluated.

## Materials and methods

### Data acquisition

The RNA-seq data of 1118 breast cancer specimens and 113 normal samples were obtained from TCGA. Data on the patient clinical features, mutations, and copy number variations (CNVs) were downloaded. These raw data were standardized at the level of fragment expression per million bases. We removed any patient data that was lacking information on patient survival. We then determined expression levels related to DRGs (Ritchie et al. 2015). When the data cleaning process was complete, we integrated the data for analysis.

### Analysis of DRGs using consensus clustering

We collected 16 DRGs from earlier published articles to form a DRG signature (Machesky 2023; Liu et al. 2023); these genes are described in Table S1. Unsupervised clustering analysis was used to separate the tumor samples into specific molecular clusters according to DRG levels, using the ConsensusClusterPlus package in R (Wilkerson and Hayes 2010). Associations between the DRGs and survival were evaluated by Kaplan–Meier curves in the “survminer” R package, and CIBERSORT was used to assess cell infiltration of the tumor tissue (Meng et al. 2020; Chen et al. 2018). Enrichment scores for immune cells were determined by gene set enrichment analysis (GSEA) (Huang et al. 2021) and were linked with clinical features. Kaplan–Meier analysis was also used to assess OS between different subtypes (*p* < 0.05).

### Construction of the prediction features of disulfidptosis-associated lncRNAs

The association between DRGs and their corresponding lncRNAs was calculated with the “limma” package R package. The levels of 125 DRG-associated lncRNAs were assessed using correlation coefficients, with *p* < 0.05 as the threshold. LncRNAs related to prognosis were identified by univariate Cox regression. The samples were randomly allocated to training and validation sets, and prognosis-linked lncRNAs were examined by LASSO regression and their predictive ability assessed using the formula: risk score = (Exp*i* × *bi*). (Exp: expression level of the model gene; *b*: model gene coefficient).

### Nomogram construction

We employed the “regplot” package to create a line chart based on clinical significance and DRG scores to predict overall survival. The line chart model was used to score each clinical significance feature, and all individual scores were summed to yield the total score. The prediction accuracy of the line chart was examined by comparing the AUCs of the time-dependent ROC curves of 1-, 3-, and 5-year survival. Calibration curves and C-indices were used for assessment of the predictive accuracy of the line chart.

### GO and KEGG analyses

Patient samples were allocated to high- and low-risk groups based on the median score. Differentially expressed genes (DEGs) were identified using FDR < 0.05 and |log2fold change (FC) > 1| as the criteria. GO and KEGG analyses were performed using the “clusterProfiler” package in R. GSEA was used to assess differences in enrichment.

### The prognostic signature and immune cell infiltration

The WGCNA algorithm was used to remove missing values, outliers, and redundancy from the data. The expression data were extracted, and the newly generated matrix was obtained by removing the censored data. Connectivities and dissimilarities in the co-expression network constructed using soft-threshold parameters were assessed with a topological overlap matrix. Co-expressed gene modules were discovered using dynamic hybrid cutting, and dynamic mixed cutting was used to construct a cluster tree where leaves represented individual genes and genes showing comparable expression or function were linked to form branches, with branches representing modules. Pearson’s correlations between genes characteristic of modules (MEs) and infiltrating immune cell types were determined, with *p* values < 0.05 indicating significant associations. Subsequently, we intersected 16 color data sets with DRGs and found that brown modular immune genes were highly correlated with DRGs. The proportions of infiltrating cells were estimated using TIMER (Li et al. 2020).

### Calculation of the TMB

The TMB is an indicator of the number of mutated per million bases and includes all types of mutations, such as nonsense, frameshift, and missense. The TMB was calculated from the variations in the total human exon length per sample using Perl scripts (Chan et al. 2019). Waterfall plots were produced using “maftools” in R to determine the point mutation numbers in the samples and the associations between TMB and risk (Mayakonda et al. 2018). Gistic2.0 was used to analyze the CNV data. Chromosomal fragments with significant numbers of amplifications and deletions were determined, and CNVs on the chromosomal arms were compared. The chromosomal positions of genes were visualized with “RCircos” in R.

### Genomic variations in the different groups

The TMB of each sample was evaluated according to the model score. In addition, we performed an analysis of somatic variant and mutation data in breast cancer patients using “maftools” (Mayakonda et al. 2018), which was also used to assess differences in mutation numbers between the groups. Chi-square tests compared the differences, which were visualized using R.

### Assessment of drug sensitivity

Chemosensitivity was predicted using data from the Genomics of Drug Sensitivity in Cancer database. Sensitivity to various drugs, including cisplatin, paclitaxel, and gemcitabine, was analyzed using the semi-maximum inhibitory concentration index.

### Statistical analysis

Data were analyzed using R v.4.2.2. Continuous variables were compared by independent *t* tests. Non-normally distributed variables were compared by Wilcoxon rank-sum tests and Chi-square tests for used for comparison of categorical variables. Patient survival was analyzed by Kaplan–Meier curves and log-rank tests. *p* values < 0.05 were considered statistically significant.

## Results

### Identification of DRGs

**Fig. 1:**
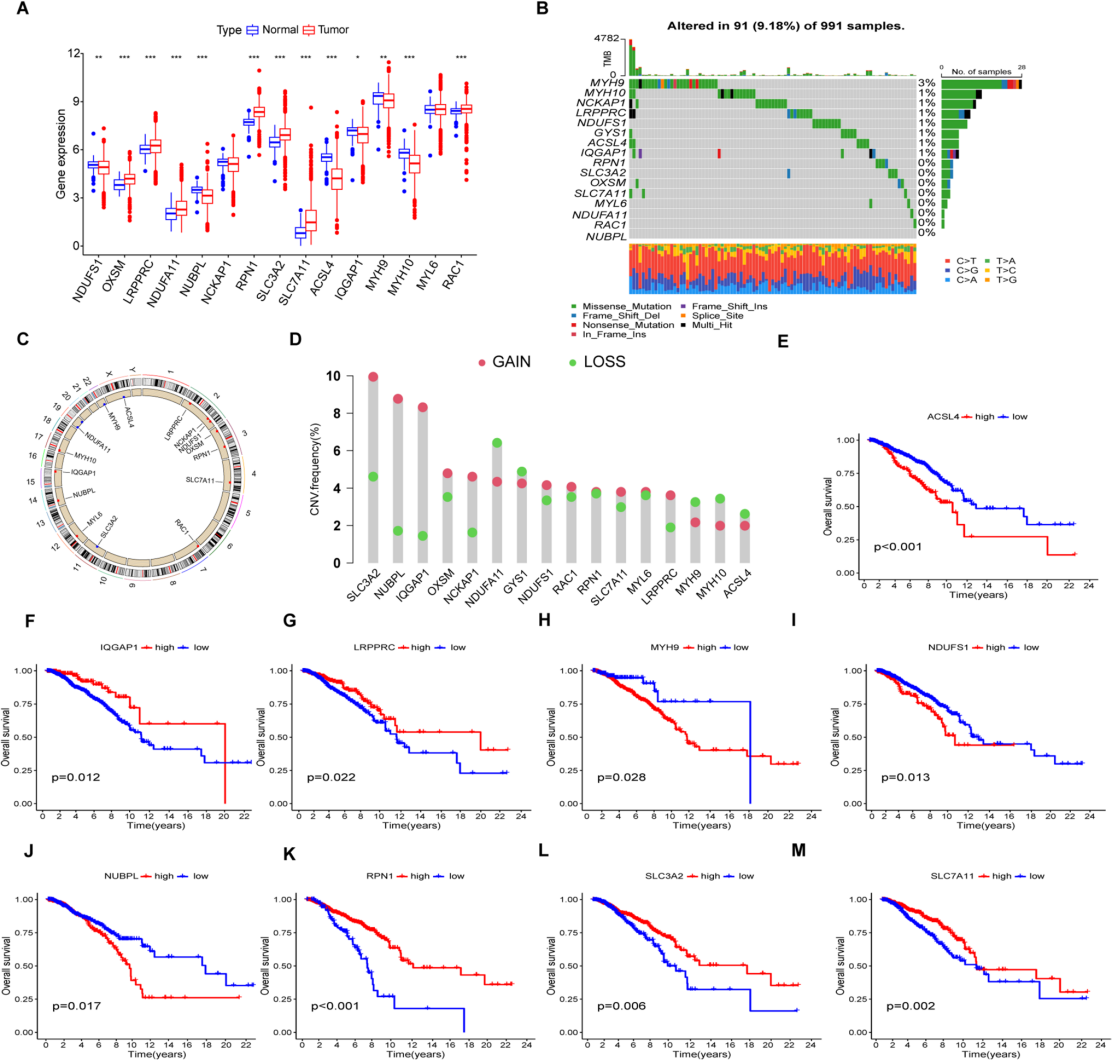
Analysis of the levels of 16 DRGs identified from TCGA data. **A** Comparison of levels of 16 DRGs between tumor and control tissues (*p < 0.05, **p < 0.01, ***p < 0.001). **B** Frequencies of DRG mutations in 991 breast cancer patients. **C** CNV variation sites of DRGs on the chromosomes. **D** Gain-of-function, loss-of-function, and neutral CNVs in DRGs. **E**–**M** Relationship between nine DRGs and OS

Sixteen DRGs were identified between 1118 tumor and control tissues (Fig. 1A). CNVs, which may lead to the activation or inactivation of genes associated with tumorigenesis (Shlien and Malkin 2009; Clifford et al. 2010), were analyzed, together with frequencies of gene amplification and deletion in the DRGs. It was found that 91 (9.18%) of the 991 samples had DRG mutations, with MYH9 showing the highest number (Fig. 1B). Most of the DRGs showed high rates of CNV deletion or accumulation. The SLC3A2, NUBPL, IQGAP1, NCKAP1, NDUFS1, RAC1, RPN1, SLC7A11, MYL6, and LRPPRC genes showed higher numbers of gain-of-function mutations, while NDUFA11, GYS1, MYH9, MYH10, and ACSL4 were associated with loss-of-function mutations (Fig. 1C, D), suggesting their relevance for breast cancer tumorigenesis.

Kaplan–Meier analysis demonstrated that increased levels of ACSL4, MYH9, NDUFS1, and NUBPL were linked with poor OS, while increased expression of IQGAP1, LRPPRC, RPN1, SLC3A2, and SLC7A11 was related to good OS (Figure levels and patient OS), suggesting the potential of these genes as targets or biomarkers for predicting outcomes in these patients.

**Fig. 2:**
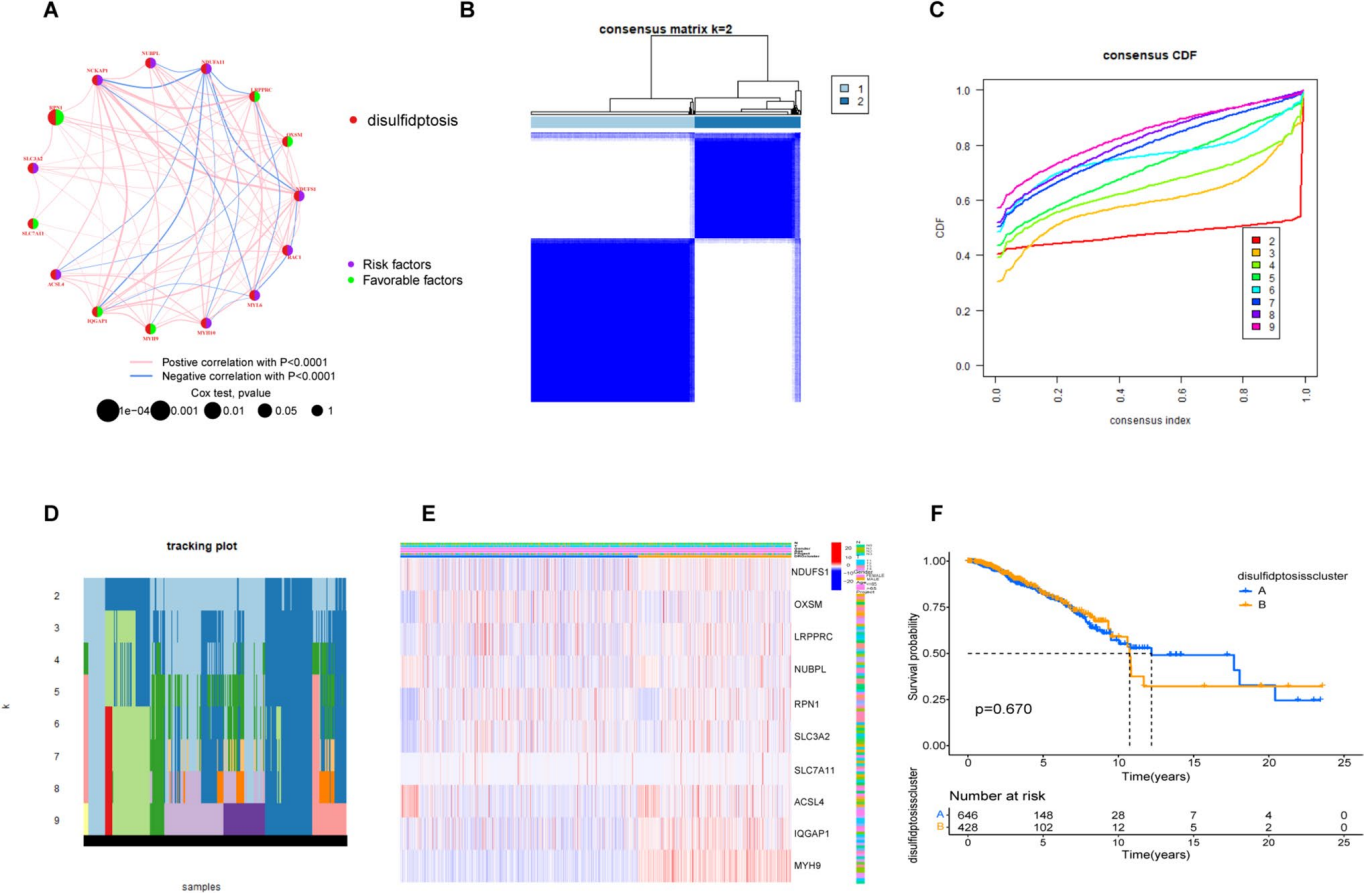
Association of DRG subtypes with patient clinical and pathological features. **A** Correlations between DRGs (red indicates positive and blue negative correlations, with greater color intensity indicative of stronger correlation). **B** Consensus matrix heatmap of the two clusters (*k* = 2). **C** The CDF is the relative change in the area under the CDF curve when *k* = 2–9. **D** The tracking plot provides clustering clusters for each sample in cases where the number of clusters is *k* = 2–9. **E** Levels and clinical pathological characteristics of DRGs in different clusters. **F** Kaplan–Meier curves of different clusters in the DRGs

The correlation network picture (Fig. 2A) shows a strong correlation between 16 DRGs. Unsupervised consensus analysis of DRG co-expression was conducted for further exploration of heterogeneity in the genes. Overall, 1231 genes were assigned to subgroups based on clinical characteristics. It was found that clusters A (*n* = 754) and B (*n* = 477) had the greatest intra-group and inter-group differences (Fig. 2B–D). The heatmap indicates the relationships between specific clinicopathological features and the two clusters. The subgroups were found to differ markedly in tumor stage and pathological grade (Fig. 2E). However, OS showed no significant difference between the subgroups (Fig. 2F) nor were differences in OS observed in terms of DRG expression. We then further analyzed the lncRNAs associated with the DRGs.

### Predictive abilities of disulfidptosis-associated lncRNAs

**Fig. 3:**
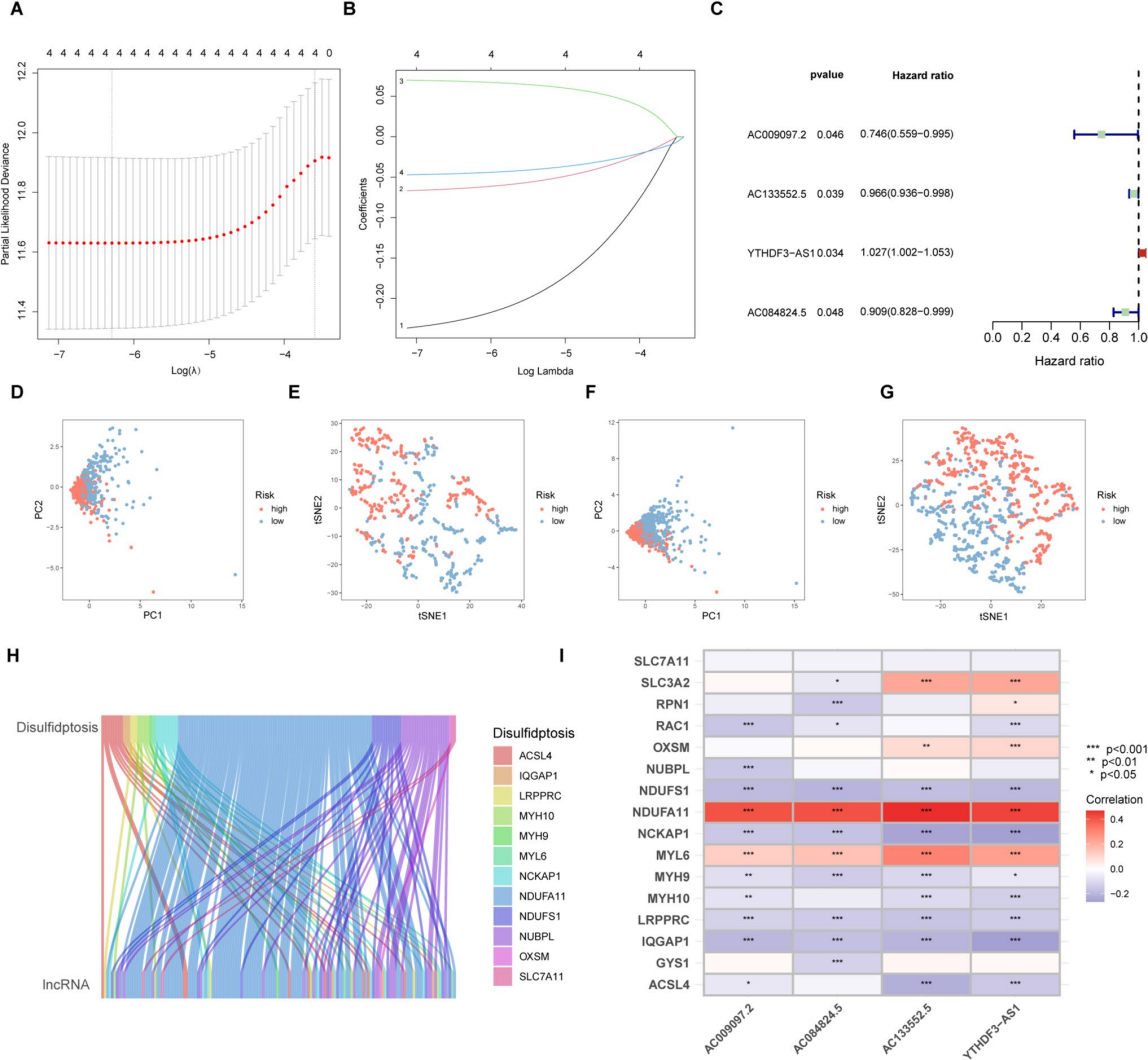
The disulfidptosis-related gene risk score (DRG-RS) model. **A** Partial likelihood deviance shown by LASSO regression with tenfold cross-validation. The vertical dotted lines represent optimal values drawn according to minimum and 1-SE criteria. **B** Four DRG-associated lncRNAs associated with prognosis. **C** Forest plot of HRs (95% CI) and *p* values for selected DEGs, identified by univariate analysis. **D**, **F** PCA of the high- and low-risk groups in the training and validation cohorts. **E**, **G** t-SNE analysis of high- and low-risk groups in the training and validation cohorts. **H** Sankey diagram showing links between DRG mRNA and lncRNAs. **I** Heatmap showing correlations between DRGs and lncRNAs

One hundred and twenty-five DRG-associated lncRNAs were identified. Overfitting was eliminated by regression analysis, which also identified prognosis-associated lncRNAs. Half of the 1231 samples were included in the training set for model construction. This was termed the disulfidptosis-related gene risk score (DRG-RS) signature and was validated with the overall TCGA dataset (Fig. 3A, B). Four lncRNAs (AC009097.2, AC133552.5, YTHDF3-AS1, and AC084824.5) were associated with prediction. AC009097.2, AC133552.5, and AC084824.5 were found to be protective and YTHDF3-AS1 was associated with increased risk (Fig. 3C). PCA and t-SNE analyses revealed that the DRG-RS model had good discriminative ability in both sets (Fig. 3D–G). In addition, the relationship between DRG-related lncRNAs was analyzed (Fig. 3H, I). We selected the following formula as the risk scoring formula: (− 0.746 * AC009097.2 expression) + (− 0.966 * AC133552.5 expression) + (1.027 * YTHDF3-AS1 expression) + (− 0.909 * AC084824.5 expression). Risk scores for individual patients were determined using this formula and the median calculated and used for sample classification according to risk.

### Disulfidptosis cell signature for prognosis prediction

**Fig. 4:**
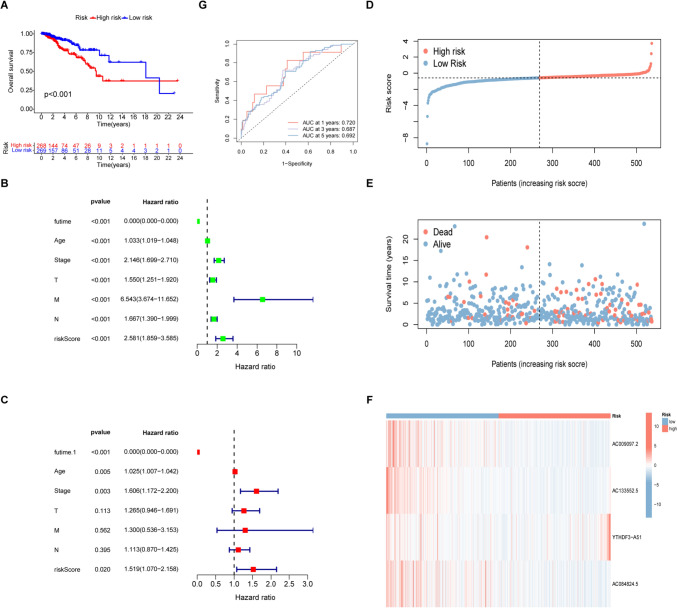
Relationships between prognosis and predictive factors. **A** Kaplan–Meier analysis of OS in the high- and low-risk groups. **B**, **C** Univariate and multivariate regression indicating the ability of the DRG-related risk score to independently predict prognosis in data from TCGA. **D**–**F** Heatmap showing risk scores, OS, and levels of genes in the two groups. **G** ROC curves for 1-, 3-, and 5-year survival in the TCGA dataset (AUC: 0.720, 0.687, 0.692)

Kaplan–Meier analysis of OS in the two groups indicated markedly reduced OS in the high-risk group (*p* < 0.001, Figs. 4A, S1). Univariate regression showed that patient age, T, N, and M stages, and the risk score model were significantly linked with OS, shown in the forest plot (Fig. 4B), while multivariate analysis indicates that the risk score, age, and stage independently predicted OS (Fig. 4C), with poor outcomes markedly related to high-risk score. There were marked differences in OS and DRG-RS distribution between the two sets (Figs. 4D–F, S2). The AUCs of the 1-, 3-, and 5-year OS in the training set were 0.720, 0.687, and 0.692, respectively, demonstrating the good predictive ability of the model (Figs. 4G, S3).

### Prognostic ability of DRG-RS and nomogram construction

**Fig. 5:**
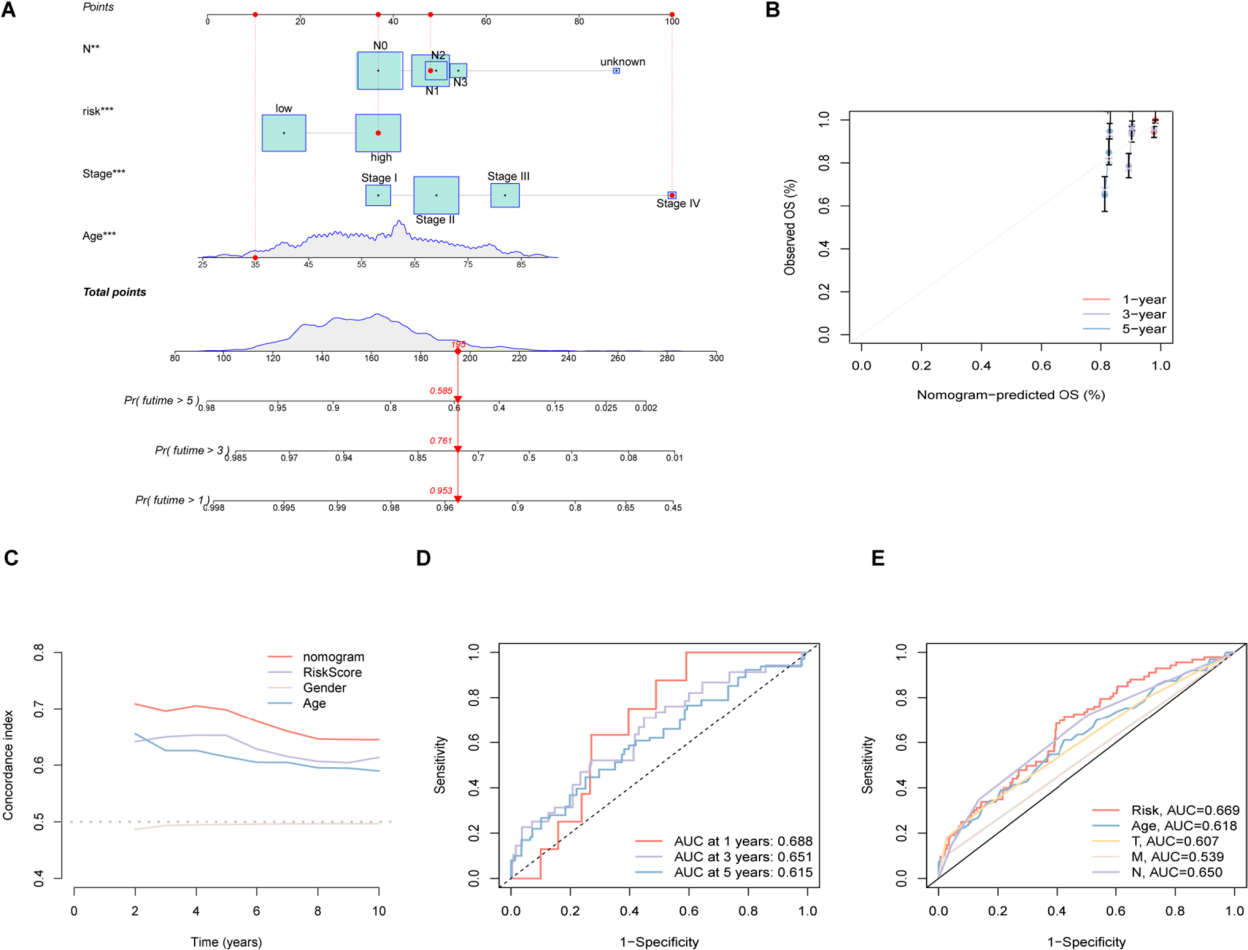
Establishment and assessment of the nomogram. **A** Nomogram construction based on risk scores and clinical features. **B** Calibration curve. **C** ROC curves of the nomogram and clinical features demonstrating superior prediction of prognosis. **D** ROC curves for 1-, 3-, and 5-year outcomes. **E** ROC curves for comparison of the predictive ability of the model with clinicopathological indicators

A nomogram was constructed to provide a quantitative method for assessing likely survival outcomes (1-, 3-, and 5-year survival) in patients with breast cancer (Fig. 5A). The total score for a patient comprised various prognostic features, including age, stage, risk score, and N stage. Poor outcomes were linked to the overall scores of the patients. The calibration curve (Fig. 5B) indicated that the observed and predicted survival outcomes were strongly consistent in the training set. Furthermore, the AUC values were all found to be above 0.65, and the nomogram of the C-index was markedly better than others, indicating that the line map based on the DRG-RS signature had superior accuracy (Fig. 5C–E).

### Functional analysis of DRG-RS

**Fig. 6:**
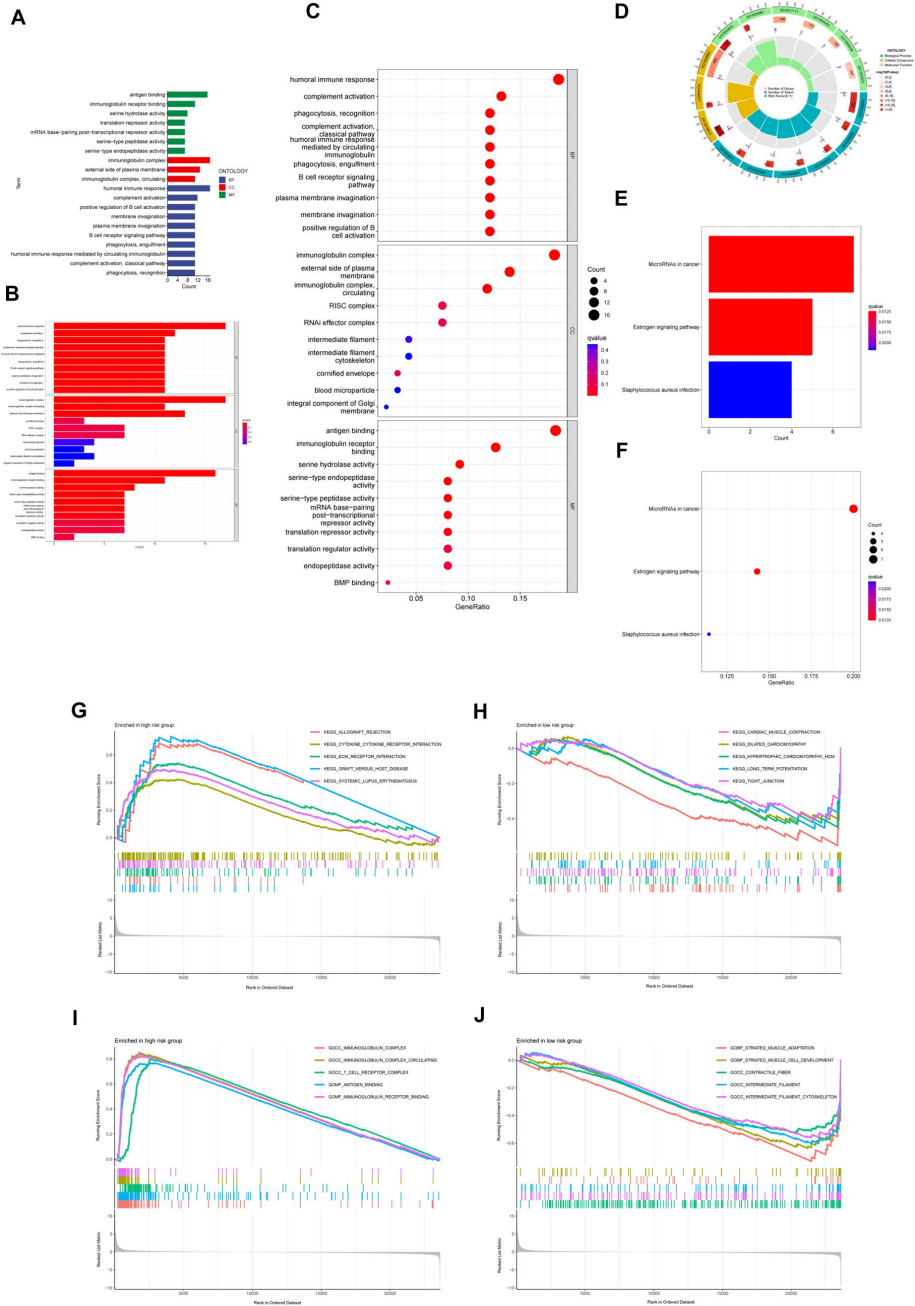
Functional analysis of DEGs between the high- and low-risk groups based on the TCGA-BRCA dataset. **A**, **B** Risk scores according to DRG-related clustering, showing DEGs between the high- and low-risk groups on a GO pathway bar plot. **C** Bubble plot; **D** circular plot. Risk scores according to DRG clusters with DEGs shown on KEGG pathway bar plot (**E**) and bubble plot (**F**). **G** GO analysis of the high-risk group. **H** GO analysis of the low-risk group. **I** KEGG analysis of the high-risk group. **J** KEGG analysis of the low-risk group

DEGs were identified between the two groups, and GO and KEGG enrichment analyses were conducted. In the GO analysis, the biological processes were mostly associated with “the humoral immune response”, “complement activation”, “phagocytosis, recognition”, “complement activation”, “the classical pathway”, and “those mediated by circulating immunoglobulin” (Fig. 6A–D). Enrichment in the cellular component category was associated with “the immunoglobulin complex”, “the external side of the plasma membrane”, “the RISC complex”, and “the RNAi effector complex”. KEGG analysis showed that the DEGs were mostly enriched in pathways related to “microRNAs in cancer”, “estrogen signaling”, and “*Staphylococcus aureus* infection” (Fig. 6E, F).

GSEA demonstrated the greatest enrichment in pathways associated with “allograft rejection”, “cytokine receptors or interaction”, “ECM receptor interaction”, “graft versus host disease”, and “systemic lupus erythematosus” in association with high risk, while low risk was associated with “cardiac muscle contraction”, “dilated cardiomyopathy”, “hypertrophic cardiomyopathy (HCM)”, “long-term potentiation”, and “tight junctions” (Fig. 6G, H). In addition, GO analysis showed enrichment in the high-risk group with “the immunoglobulin complex”, “circulating immunoglobulin complex”, “antigen-binding”, and “immunoglobulin receptor binding” (Fig. 6I), while pathways such as “striated muscle adaptation”, “striated muscle cell development”, “contractile fibers” “intermediate filaments”, and “the intermediate filament cytoskeleton” were linked with low risk(Fig. 6J).

### Clinicopathological features and the candidate lncRNAs

**Fig. 7:**
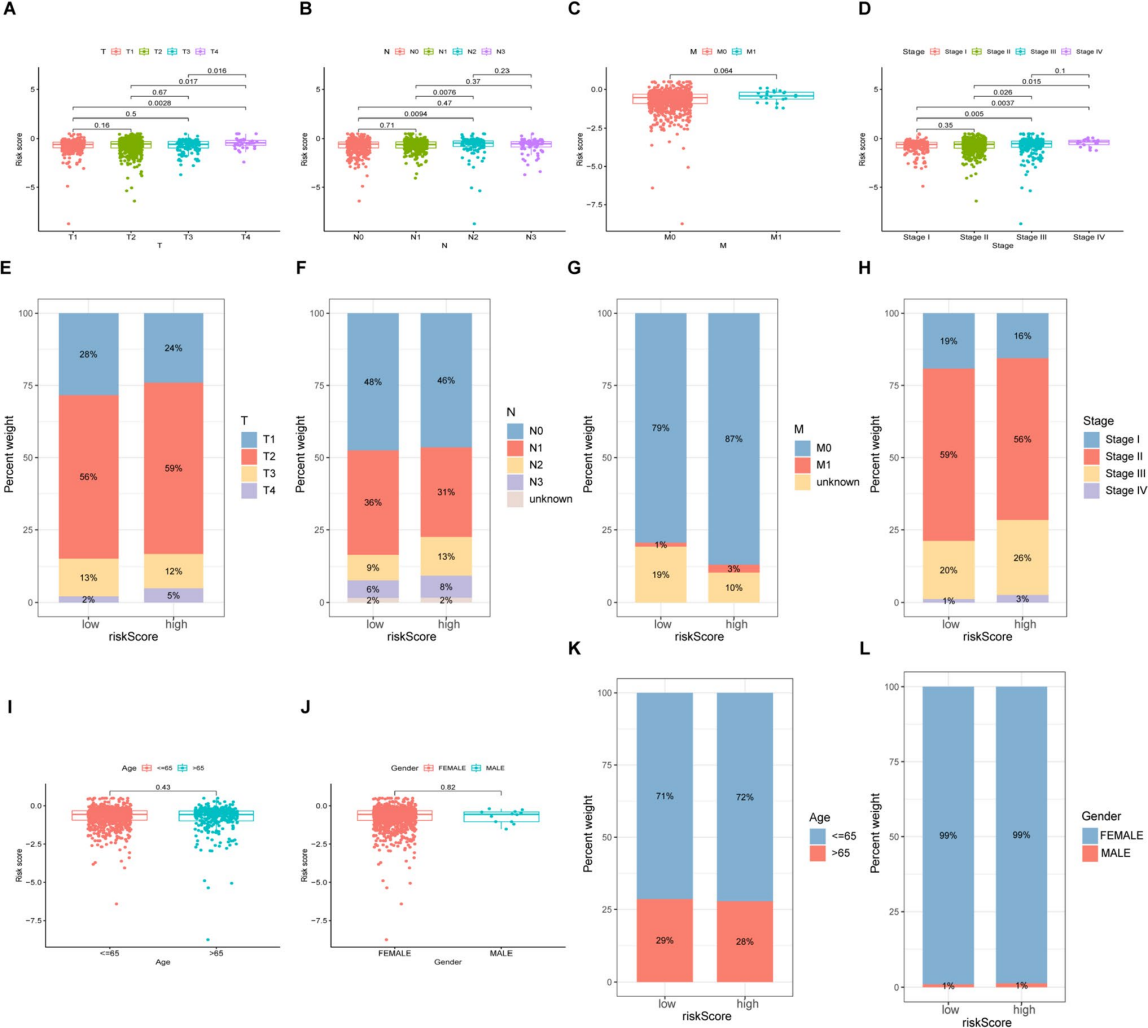
DRG-associated and clinicopathological features of patient from the TCGA dataset. **A**–**C** Boxplot showing DRG-based risk scores in patients with different T, N, and M stages. **D** Boxplot showing DRG-based risk scores in relation to stage. **E**–**H** Distributions and percentages of T, N, and M stages in the two groups. **I**, **J** Boxplot showing DRG-associated risk scores in relation to age and sex. **K**, **L** Distributions and percentages of age and sex in the two groups

The scatter plot (Fig. 7A–D) shows that the high- and low-risk groups differed in TNM, stage, and risk score. Differences in the T level were as follows: T1 28% vs 24%, T2 56% vs 59%, T3 13% vs 12%, and T4 2% vs 5%, respectively. Differences in the N level were as follows: N0 48% vs 46%, N1 36% vs 31%, N2 9% vs 13%, and N3 6% vs 8%, respectively. Differences in the M level were as follows: M0 79% vs 89% and M1 1% vs 3%, respectively), demonstrating that high-risk that patients tended to have increased TNM grades and stages (Fig. 7E–H). However, age and sex were similar in both groups (Fig. 7I–L).

### Associations of DRGs with immune cell infiltration

**Fig. 8:**
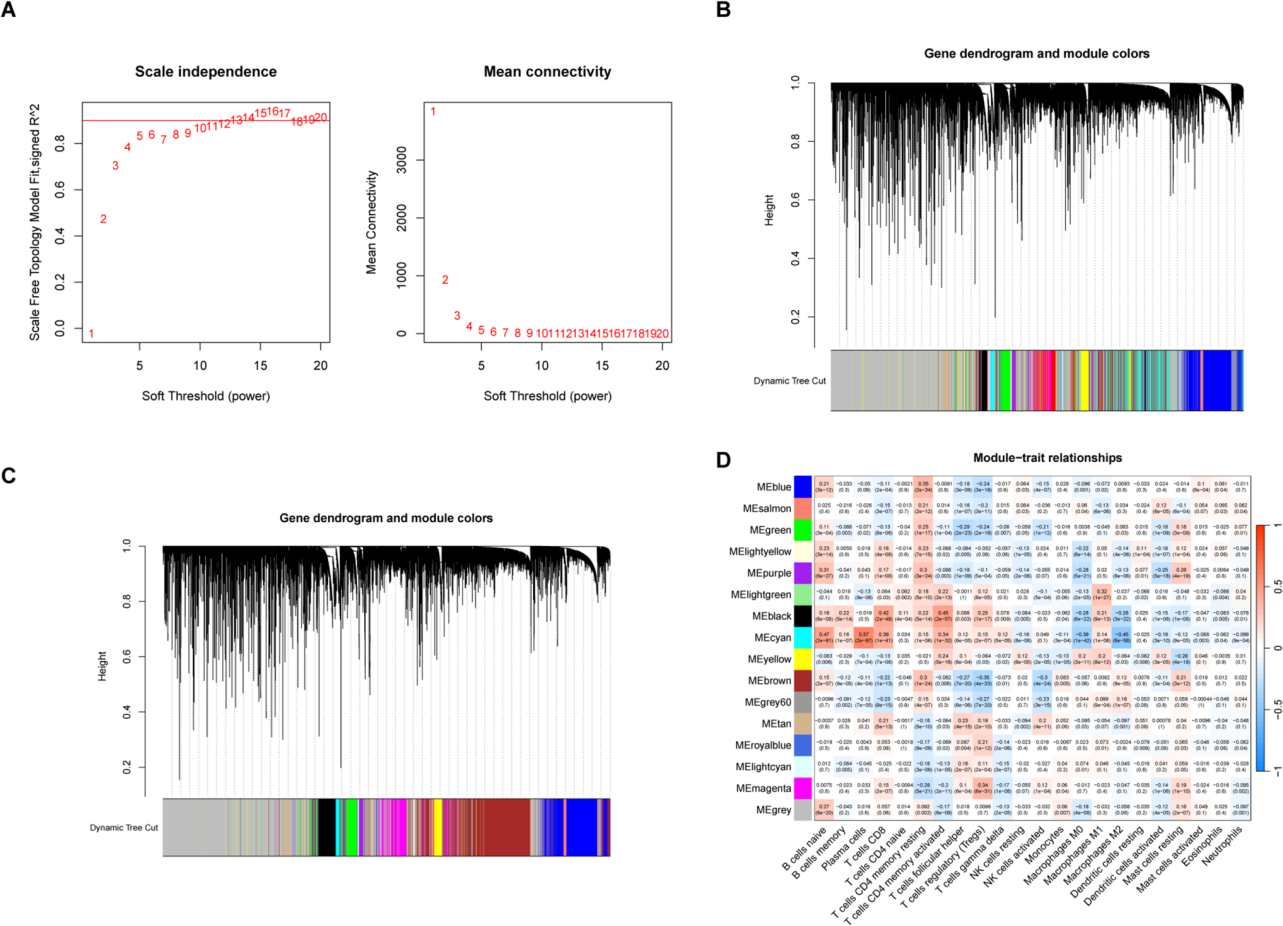
Co-expression network constructed by WGCNA. **A** Scale independence and mean connectivity analysis for various soft threshold powers. **B** A Hierarchical clustering tree constructed based on RNA-seq. **C** Merging of similar functional modules to construct a new hierarchical clustering tree. **D** Module–trait relationships. Rows represent module eigengenes and columns represent traits. Each cell includes the corresponding correlation and *p* value

A co-expression module was developed to show the relationship between immune-related genes and sample characteristics using WGCNA. This involved the compilation of a clustering tree, removal of abnormal samples, calculation of correlation coefficients between gene pairs, and the construction of a similarity matrix using gene expression data. The similarity matrix was then converted into an adjacency matrix using the soft threshold rule (16) (Fig. 8A). A scale-free network and topological overlap matrix (TOM) were then constructed to assess the relationships between genes, and hierarchical clustering of genes (Fig. 8B) was analyzed by a hierarchical clustering tree. Final modules were produced using the dynamic tree-cutting method (Fig. 8C). The resulting 16 modules are shown in different colors: blue, salmon, green, light yellow, purple, light green, black, cyan, yellow, brown, gray, tan, royal blue, light cyan, magenta, and gray. A Venn diagram was used for visualization of the overlap, finding that genes in the brown module were strongly linked to DRGs. In breast cancer, the module–trait correlation heat map showed a high correlation between DRGs and naive B cells (*R*^2^ = 0.15, *p* = 2e−07), resting memory T cells CD4 (*R*^2^ = 0.083, *p* = 1e−24), monocytes (*R*^2^ = 0.083, *p* = 0.005), macrophages M2 (*R*^2^ = 0.12, *p* = 9e−05), and resting mast cells (*R*^2^ = 0.21, *p* = 3e−12) (Fig. 8D). This suggests that DRGs are highly correlated with immune infiltration.

### Immune infiltration in the two groups

**Fig. 9:**
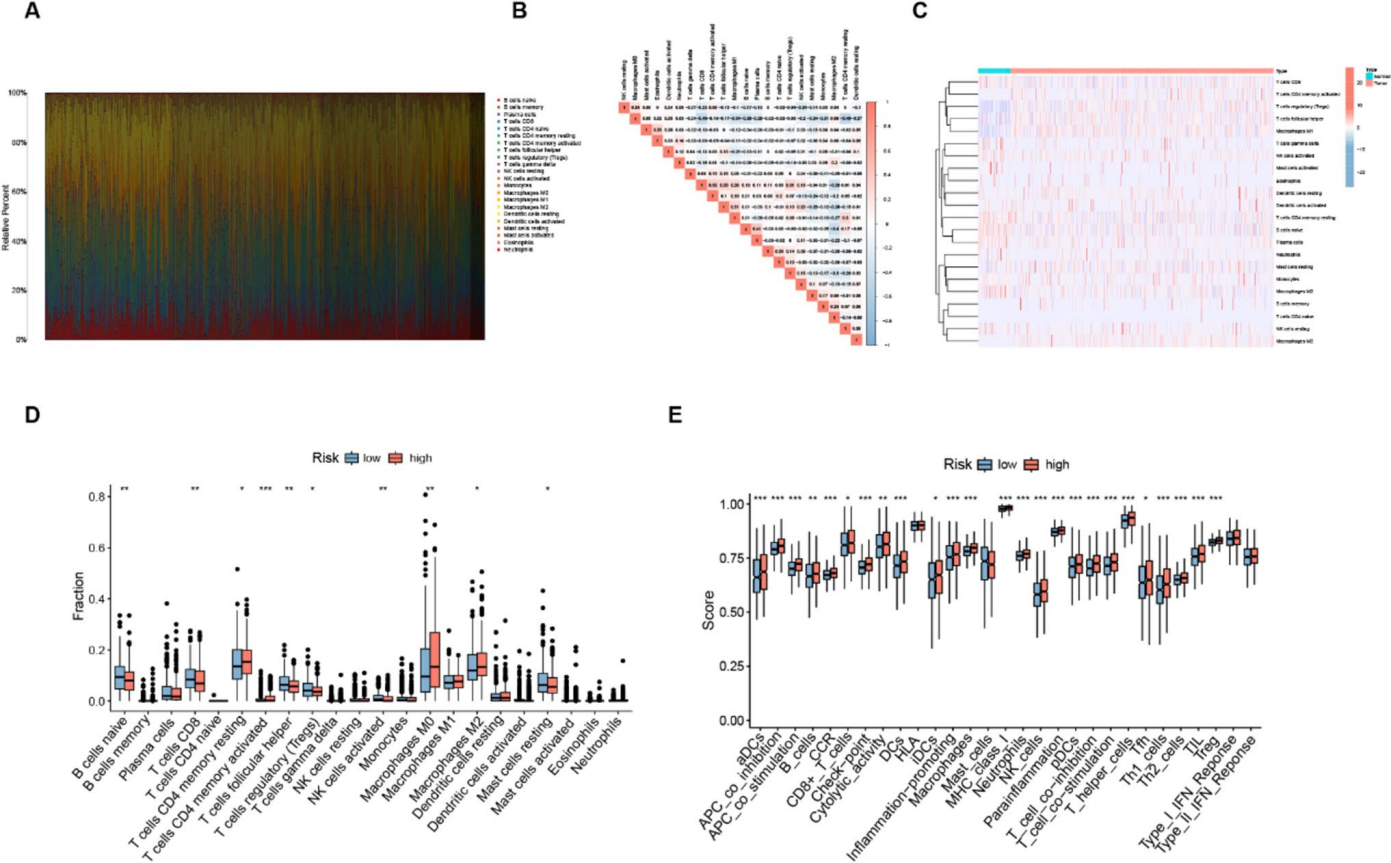
Immune cell infiltration in relation to the DRG-RS in the TCGA dataset. **A** Relative proportions of 22 immune cell types in relation to high and low DRG-RS. **B** CorHeatmap of infiltrating immune cells. **C** Expression heatmap of 22 infiltrating immune cell types in tumor and control tissues from TCGA. **D** Association between immune cell types and risk groups (***p < 0.001, **p < 0.01, *p < 0.05). **E** Analysis of immune signatures in high- and low-risk groups, shown by GSEA

The relative abundances of 22 immune cell types in relation to DRG-RS subtype were investigated using CIBERSORT. Figure 9A shows the abundances of immune cells in the DRG-RS subtypes, while Fig. 9B shows the correlations between immune cell types and the TCGA groups (Fig. 9B). The R package “limma” R was used to compare the abundance of immune cell subsets and associated functions. Differences were observed in naive B cells, CD8 + T cells, resting memory CD4 T cells, activated memory CD4 T cells, T follicular helper cells, Tregs, activated NK cells, M0 and M2 macrophages, and resting mast cells between the two groups (*p* value < 0.05, Fig. 9C). The cell types and functions were associated with greater activity in the high-risk group and included aDCs, B cells, CCR, CD8 + T cells, macrophages, T helper cells, iDCs and pDCs, NK cells, Tregs, TIL cells, Tfh, Th1, and Th2 cells, and pathways associated with APC co-inhibition and co-stimulation, checkpoints, T cell co-inhibition and co-stimulation, cytolytic activity, and the promotion of inflammation and para-inflammation (Fig. 9D). This suggests that the immune processes of the high-risk group are relatively more active than those in the low-risk group.

### Relationship between TMN and DRG-RS

**Fig. 10:**
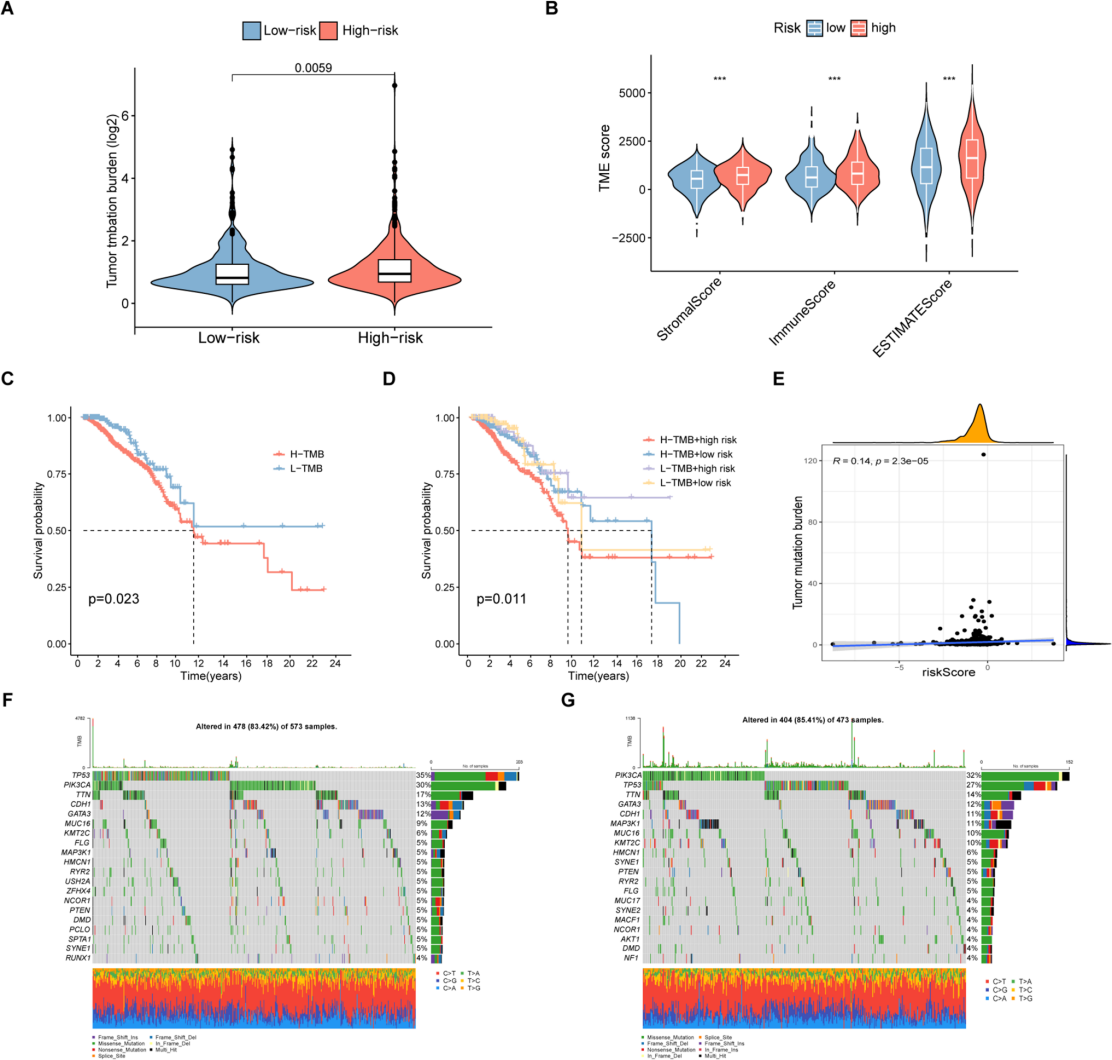
Relationship between TMB and DRG-RS. **A** Violin plot of TMB in the high- and low-risk groups. **B** Immune, stromal, and ESTIMATE scores. **C** TMB and risk scores in the stratified patient Kaplan–Meier curves. **D** Kaplan–Meier curve stratified by TMB group and DRG signature. **E** Correlation between DRG risk and TMB scores in the TCGA dataset. Waterfall plot of mutations in the high- (**F**) and low-risk (**G**) groups

The TMB is an indicator of mutation numbers and can be related to T cell recognition (Pitt et al. 2016; Vinay et al. 2015; Herbst et al. 2018; Altorki et al. 2019). Therefore, we inferred that TMB may be a prognostic indicator that should not be ignored in anti-tumor immunotherapy and thus investigated relationships between TMBs and risk scores. Positive associations were seen between the DRG-RS and TMB (*R* = 0.14, *p* = 2.3e−05, Fig. 10E), with TMB values being linked with high risk (*p* = 0.0059, Fig. 10A). The immune, stromal, and ESTIMATE scores were assessed using ESTIMATE (Fig. 10B) (Yoshihara et al. 2013). The associations between the combined TMB and DRG risk scores on OS were then assessed, finding that high TMB was associated with poorer prognosis and that there was a synergistic effect between TMB and the DRG-RS (Fig. 10C, D). The waterfall chart indicates the distribution of mutations in the top genes in the groups. The genes showing the greatest number of mutations were TP53, PIK3CA, TTN, CDH1, GATA3, MUC16, KMT2C, FLG, MAP3K1, and HMCN1. In high-risk patients, TP53 (35%) and PIK3CA (30%) (Fig. 10F) had greater mutational rates, while PIK3CA (32%) was higher in those with low risk (Fig. 10G). These results may offer clues for distinguishing relationships between risk and somatic variation in breast cancer immunotherapy, as well as assisting the interpretation of the immunotherapy response (Jiang et al. 2022).

### Drug sensitivity of DRGs in breast cancer

**Fig. 11:**
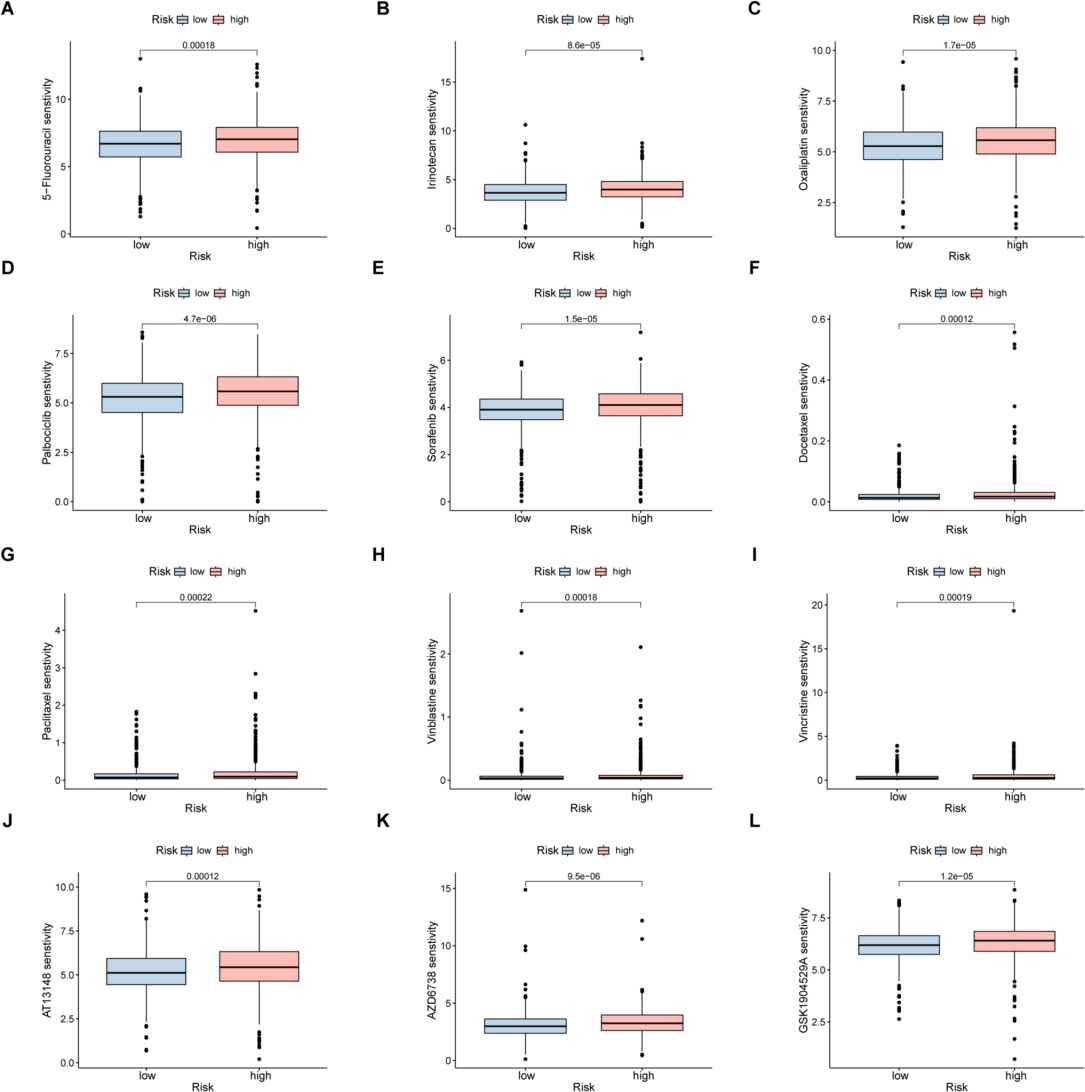
Sensitivity to chemotherapy drugs in the different risk groups

The GDSC database was used for identification of chemotherapy drugs for analysis of drug sensitivity. It was observed that the IC50 values of 12 chemotherapy drugs were considerably reduced in the high-risk group, namely 5-fluorouracil, irinotecan, oxaliplatin, palbociclib, sorafenib, docetaxel, paclitaxel, vinblastine, vincristine, AT13148, AZD6738, and GSK1904529A (*p* < 0.05, Fig. 11A–P). This indicates that high-risk patients may benefit the use of these drugs and that DRG activation may enhance drug sensitivity in breast cancer patients.

## Discussion

Disulfidptosis results from stress caused by abnormal amounts of disulfide bonds in the actin components of the cytoskeleton, leading to the collapse of F-actin (Machesky 2023) and cytotoxicity (Liu et al. 2020; Joly et al. 2020). As this form of cell death has only recently been discovered, there is minimal information on its role in breast cancer. This disease is a significant public health issue, with approximately 2.26 million diagnoses (11.7%) annually (Sung et al. 2021; Ahmad 2019; Liu et al. 2021). It is now the most common female malignancy and its incidence is increasing (Hutchinson 2010). Thus, the identification of biomarkers for both diagnosis and outcome prediction is most important (Weigel and Dowsett 2010; Shi et al. 2022). Here, a prognostic risk model was developed according to univariate Cox regression and LASSO analyses to elucidate the possible association between disulfidptosis and breast cancer outcomes.

Significant differences in DRG levels were found between cancer tissues and controls (*p* < 0.05). This led to the speculation that DRG levels may assist in the prediction of breast cancer prognosis. A DRG signature was identified by regression analyses while excluding overfitting effects. This signature, together with the clinical features of the patients, showed good predictive value for patient survival. The AUCs of the ROC curves were used to examine 1-year (0.720), 3-year (0.687), and 5-year (0.692) survival, demonstrating the effectiveness of the model for the prediction of patient prognosis. Samples were classified as low or high risk. These did not take into account the breast cancer molecular subtype. The OS values in high-risk samples were observed to be markedly reduced in comparison with low-risk samples. It was also found that patients in the high-risk group showed greater activity of infiltrating immune cells, together with greater numbers of somatic mutations, and significantly different drug sensitivities than their low-risk counterparts.

Tumorigenesis and tumor development jointly represent a complex process. Cancer cells interact dynamically with both the immune system and the tumor microenvironment (TME) (Chen and Mellman 2017). The function of the TME in tumor development, progression, and reduced drug sensitivity is well-documented (Sathe et al. 2020; Galvani et al. 2020; Huang et al. 2020). Tumor-infiltrating immune cells (TIICs) have also been implicated in prognostic improvement in breast cancer, as well as in the mediation of the response to both immunotherapy and chemotherapy. Here, WGCNA analysis showed that DRG signature scores were strongly linked with TIIC abundance in tumor samples, demonstrating the effectiveness of the DRG-RS model in identifying the abundance of 22 TIICs in the TME.

Several TIIC types were observed to be more abundant and active in the high-risk group in comparison with the low-risk group. These included M0, M1, and M2 macrophages, resting dendritic cells, naive B cells, CD8 + T cells, and resting CD4 memory T cells (*p* < 0.05). As shown by earlier studies, TME-associated cells may both enhance or restrict tumor growth (Miguel and Calvo 2020; Yan et al. 2020; Ye et al. 2021), dependent on the type of tumor, cell ontogeny, and levels associated with both the tumor itself and the body overall; all these factors can influence drug sensitivity or resistance (Junttila and Sauvage 2013; Klemm and Joyce 2015; Binnewies et al. 2018). TIICs also induce tumor cell invasion (DeNardo and Ruffell 2019; Mantovani et al. 2017), which explains the association between increased numbers of tumor-associated macrophages (TAMs) and tumor-infiltrating dendritic cells (TIDCs) with poor outcomes in patients with breast cancer. The findings of an earlier study confirm our present observations that high risk was linked to increased infiltration of TIICs, poor OS, and greater resistance to chemotherapy drugs. The DRG-RS model could be effective for determining survival prognosis in these two patient groups. The identification of additional prognostic biomarkers for the model together with the TME may lead to the identification of new targets for breast cancer treatment.

Chemotherapy is currently a major strategy used in breast cancer. It is thus important to discover effective drugs for treating this disease (Denkert et al. 2018; Pruneri et al. 2018; Ali et al. 2016; Foukakis et al. 2018; Weiss et al. 2018). Unfortunately, both tumor heterogeneity and drug resistance have resulted in reduced responses to chemotherapy. The different DRG-RS risk groups responded differently to drugs used in conventional chemotherapy. We, therefore, speculate that specifically targeted treatment for different patient groups may prove to be more effective. It is worth noting that the IC50 values for the frequently used chemotherapy drugs docetaxel, paclitaxel, and oxaliplatin were found to be lower, resulting in greater sensitivity and thus efficacy in high-risk patients. This finding contrasts with the overall poor prognosis for high-risk patients, suggesting that patient response to drugs is not necessarily key to prognosis. According to the findings of this study, we were pleasantly surprised to find that disulfidptosis appears to overcome the weaknesses of programmed cell death in cancer, which may reduce or even reverse the insensitivity of tumor cells to chemotherapeutic drugs. We speculate that tumor cell sensitivity to disulfidptosis combined with the anticancer effect of other drugs may provide a new means to develop novel and effective cancer treatments. Of course, this hypothesis requires further verification.

Here, the purpose was to examine the possible association between DRGs and prognosis in patients with breast cancer and to construct a novel and innovative DRG-RS model. Using multi-angle exploration and verification, it was found that the resulting DRG-RS model has good potential for prognosis prediction. Nevertheless, despite the good performance of the model in both cohorts, there are still several limitations. First, the data were obtained from a single database and may thus be subject to data bias and also be lacking in some important clinical details, such as grade classification, adjuvant chemotherapy, and other patient information; thus, the effects of these factors could not be explored. Second, the DRG-RS model requires verification using a prospective, multicenter study with a larger sample size. Third, high-quality multicenter randomized controlled trials are necessary, with large sample sizes and full follow-up information for additional verification and clarification of the mechanism underlying the role of DRGs in breast cancer.

## Conclusion

Here, the functions of DRGs in the prognosis of breast cancer were systematically analyzed, and the associations between the TMB, TME, and clinical characteristics were used for the establishment of a prognostic model. Furthermore, the effectiveness of the DRG signature as a marker of likely therapeutic response was evaluated. In summary, the findings reveal the clinical importance of DRGs and provide a foundation for future research.

## Supplementary Information

Below is the link to the electronic supplementary material.

Additional file 1:Figure S1 Kaplan–Meier curves of OS in the high- and low-risk groups in the testing set. Additional file 2:Figure S2 The relationship between prognosis and predictive characteristics of breast cancer patients. The risk score distribution (A), survival status distribution(B), and gene expression heatmap(C) of breast cancer patients in the high-risk and low-risk groups of the testing set. Additional file 3:Figure S3 ROC curves for 1-, 3-, and 5-year survival in the testing set.(AUC: 0.702, 0.669, 0.657). Additional file 4: Table S1 Correlation of disulfidptosis-related genes. Additional file 5: Table S2 Correlation of disulfidptosis-related genes with OS in BC. (PDF 485 kb)

## Data Availability

All the data corresponding to the BRCA series used in this study are available in TCGA (https://portal.gdc.cancer.gov/), which are public functional genomics data repositories.

## Abbreviations

DRGs: Disulfidptosis-related genes
LASSO: Least absolute shrinkage and selection operator
TCGA: The Cancer Genome Atlas
TMB: Tumor mutation burden
GO: Gene ontology
KEGG: Kyoto Encyclopedia of genes and genomes
ESTIMATE: Estimation of stromal and immune cells in malignant tumor tissues using expression data
CIBERSORT: Cell-type identification by estimating relative subsets of RNA transcripts
ssGSEA: Single-sample gene set enrichment analysis
WGCNA: Weighted gene co-expression network analysis
ROC: Receiver operating characteristic
AUC: Area under the curve
CNV: Copy number variation
DEGs: Differentially expressed genes
PCA: Principal component analysis
t-SNE: T-distributed stochastic neighbor embedding
TAMs: Tumor-associated macrophages
TIDCs: Tumor-infiltrating dendritic cells
TOM: Topological overlap matrix
TOM: Topological overlap matrix
TME: Tumor microenvironment
IC50: Half-maximal inhibitory concentration
