# Supplementary material for: Integrative landscape analysis of prognostic model biomarkers and immunogenomics of disulfidptosis-related genes in breast cancer based on LASSO and WGCNA analyses
Source: J Cancer Res Clin Oncol. 2023 Sep 22;149(18):16851–67. doi: 10.1007/s00432-023-05372-z (PMC10645620; doi:10.1007/s00432-023-05372-z)
Supplement: Supplementary file 1 — Additional file 1:Figure S1 Kaplan–Meier curves of OS in the high- and low-risk groups in the testing set. Additional file 2:Figure S2 The relationship between prognosis and predictive characteristics of breast cancer patients. The risk score distribution (A), survival status distribution(B), and gene expression heatmap(C) of breast cancer patients in the high-risk and low-risk groups of the testing set. Additional file 3:Figure S3 ROC curves for 1-, 3-, and 5-year survival in the testing set.(AUC: 0.702, 0.669, 0.657). Additional file 4: Table S1 Correlation of disulfidptosis-related genes. Additional file 5: Table S2 Correlation of disulfidptosis-related genes with OS in BC. (PDF 485 kb) [file 432_2023_5372_MOESM1_ESM.pdf]

Fig S1

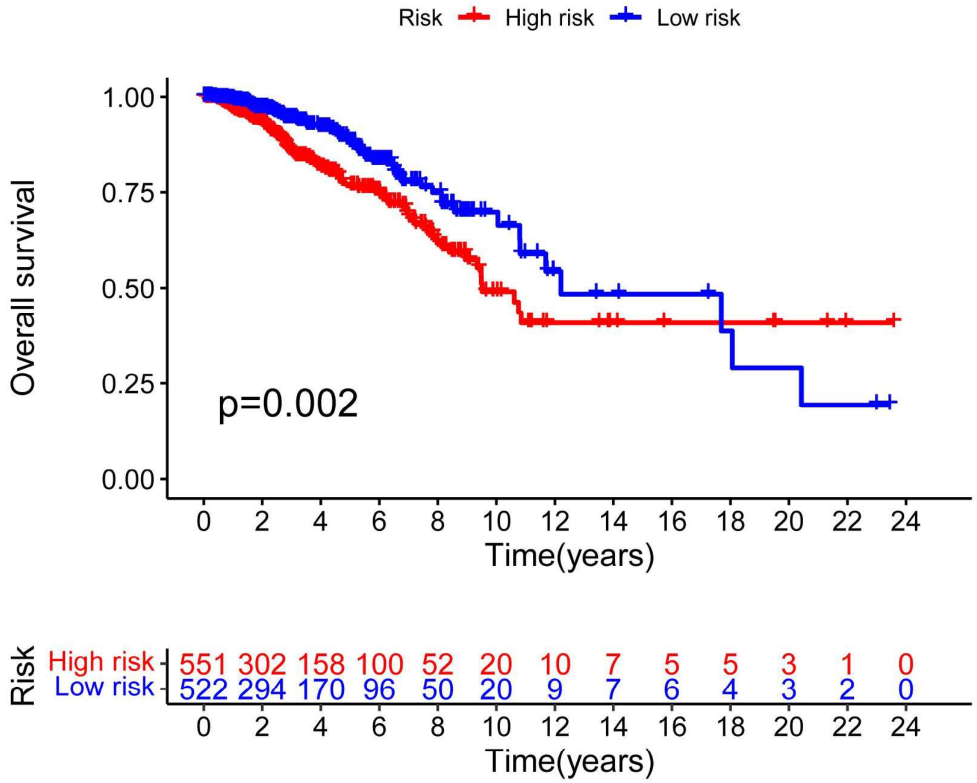
Risk
High risk
Low risk
Overall survival
1.00
0.75
0.50
0.25
0.00
p=0.002
0 2 4 6 8 10 12 14 16 18 20 22 24
Time(years)
Risk
High risk 551 302 158 100 52 20 10 7 5 5 3 1 0
Low risk 522 294 170 96 50 20 9 7 6 4 3 2 0
0 2 4 6 8 10 12 14 16 18 20 22 24
Time(years)

Fig S2

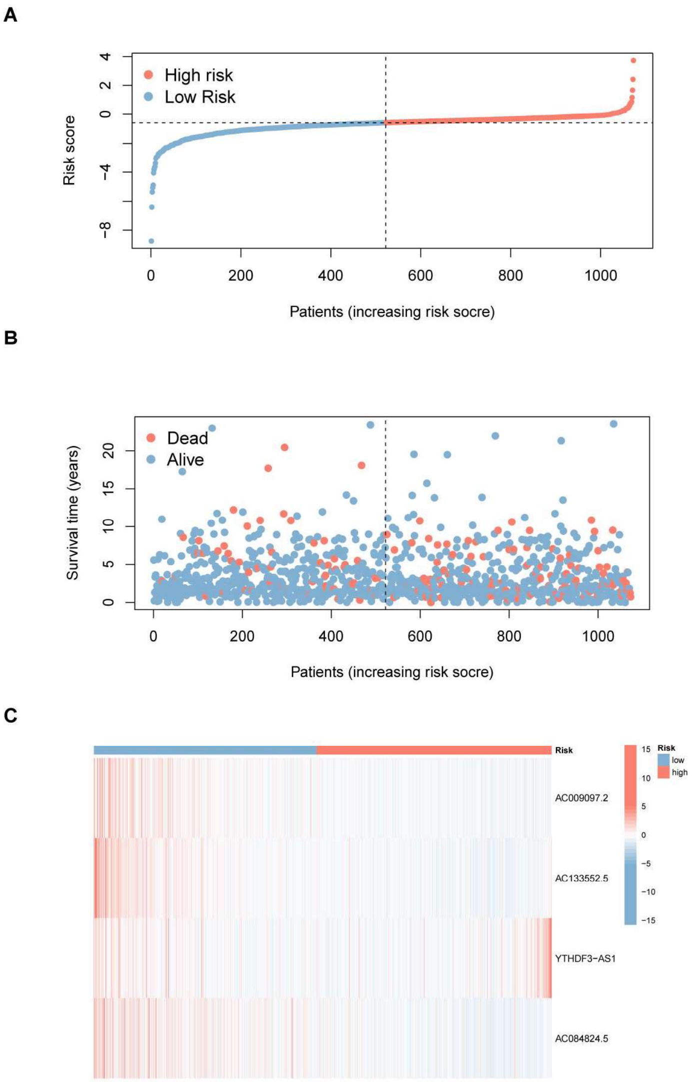
A
High risk
Low Risk
Risk score
Patients (increasing risk socre)
B
Dead
Alive
Survival time (years)
Patients (increasing risk socre)
C
Risk
AC009097.2
AC133552.5
YTHDF3−AS1
AC084824.5
low
high

Fig S3

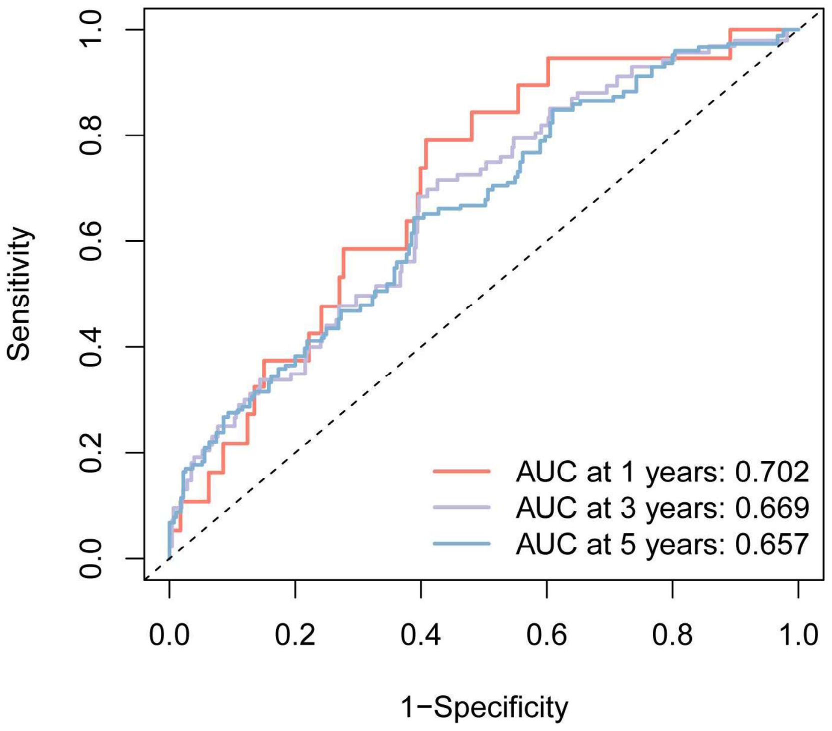

1.0
0.8
0.6
0.4
0.2
0.0
Sensitivity
AUC at 1 years: 0.702
AUC at 3 years: 0.669
AUC at 5 years: 0.657
0.0
0.2
0.4
0.6
0.8
1.0
1−Specificity

Table S1

Correlation of disulfidptosis-related genes
GYS1
NDUFS1
OXSM
LRPPRC
NDUFA11
NUBPL
NCKAP1
RPN1
SLC3A2
SLC7A11
ACSL4
IQGAP1
MYH9
MYH10
MYL6
RAC1

Table S2

Correlation of disulfidptosis-related genes with OS in BC					
id	HR	HR.95L	HR.95H	pvalue	km
**NDUFS1**	1.008883	0.997045	1.020862	0.141962	0.013384
OXSM	0.980657	0.954054	1.008002	0.163932	0.005381
LRPPRC	0.999981	0.995557	1.004425	0.993415	0.021881
NDUFA11	1.005127	0.960486	1.051843	0.825375	0.052046
NUBPL	1.017232	0.995019	1.03994	0.129348	0.017191
NCKAP1	1.00482	0.995134	1.014601	0.330542	0.100296
RPN1	0.997705	0.996169	0.999242	0.003446	7.06E-07
SLC3A2	1.000129	0.99768	1.002584	0.91765	0.005808
SLC7A11	0.999744	0.980352	1.01952	0.979577	0.001991
ACSL4	1.003654	0.997557	1.009789	0.240744	0.000384
IQGAP1	0.998431	0.995893	1.000975	0.226519	0.01236
MYH9	0.999806	0.999147	1.000466	0.564753	0.028363
MYH10	1.002887	0.995768	1.010057	0.427747	0.063191
MYL6	1.000003	0.998845	1.001161	0.996501	0.169345
RAC1	1.000702	0.999256	1.00215	0.341494	0.077133
